# Overall Weak Association Between Gastrointestinal Symptoms and Psychological, Autism, and Demographic Variables in Autistic Children

**DOI:** 10.1177/13623613261464209

**Published:** 2026-08-05

**Authors:** Eliana Rosenthal, Daniel A. Waschbusch, Susan D. Mayes

**Affiliations:** 1 Penn State College of Medicine, Hershey, USA

**Keywords:** children, autism, gastrointestinal, co-occurring symptoms

## Abstract

Our study determined psychological, autism trait, somatic, and demographic variables associated with maternal ratings of stomachaches, constipation, diarrhea, and bowel incontinence in autistic children. Mothers of 1,093 autistic children rated their children on the Pediatric Behavior Scale. In regression analyses, the somatic symptom score (i.e., headaches, other aches/pains excluding stomachaches, complains of feeling sick) was the strongest predictor of stomachaches, constipation, and diarrhea. Scores on the other variables (autism severity, externalizing symptoms, internalizing symptoms, autism traits, sleep problems, demographics) each contributed less than 2% more to explained variance. Decreasing age and decreasing IQ were predictors of bowel incontinence. Only 1 of the 30 significant correlations between GI symptoms and the independent variables was large (stomachaches and other somatic symptoms), one was medium (stomachaches and sadness), and four were small to medium (stomachaches and anxiety and increasing age; bowel incontinence and decreasing age and decreasing IQ). The association between GI problems and psychopathology, autism symptoms, and demographics is weak. Findings suggest that researchers and clinicians need to look beyond these variables and consider medical, physiological, neurobiological, and genetic reasons why GI problems are so common in autism relative to children with other neurodevelopmental disorders and neurotypical children.

**Lay Abstract:**

This study explored the reasons why autistic children often experience stomachaches and other digestive issues. We found that other physical symptoms, like headaches, were the biggest predictors of stomach problems, while factors like autism severity, age, or IQ had less influence. The researchers suggest that there may be medical or physical reasons behind these issues, beyond what is currently understood.

## Introduction

Gastrointestinal (GI) problems are common in autistic youth. In one study of 50 autistic youth, 70% had GI symptoms, compared with 42% of youth with other developmental disorders and 28% of typical children (Valicenti-McDermott et al., 2006). Other studies also report elevated rates of GI problems in autism relative to neurotypical children and those with other developmental disabilities (e.g., Babinska et al., 2020; Bresnahan et al., 2015; Chaidez et al., 2014; Gan et al., 2023; Mayes et al., 2021; Reynolds et al., 2021). A systematic review of 30 studies found contradictory evidence of relationships between GI symptoms and co-occurring conditions or other variables in autism (Leader et al., 2022), highlighting the need to clarify which factors are most strongly associated with GI problems.

Research examining autism-related traits has yielded inconsistent associations with GI symptoms. Some studies found no link between autism severity and GI problems (Babinska et al., 2020; Chandler et al., 2013; Jiang et al., 2017; Mazefsky et al., 2014; Prosperi et al., 2017; Restrepo et al., 2020; Reynolds et al., 2021), whereas others reported a positive relationship (Khalil et al., 2021; Wang et al., 2011; Yang et al., 2018). Findings for developmental regression are similarly mixed, with one study reporting more GI symptoms in children with regression (Reynolds et al., 2021) and others finding no difference (Chandler et al., 2013; Hansen et al., 2008; Molloy & Manning-Courtney, 2003). Picky eating and sensory over-responsivity have also shown variable associations, with some studies reporting significant relationships (Babinska et al., 2020; Leader et al., 2020; Mayes & Zickgraf, 2019; Mazurek et al., 2013; Valicenti-McDermott et al., 2006) and others not (Chandler et al., 2013; Gorrindo et al., 2012; Khalil et al., 2021). Externalizing behaviors (oppositionality, aggression, ADHD symptoms) and internalizing problems (anxiety, depression) have shown mixed associations with GI symptoms (Chaidez et al., 2014; Dovgan et al., 2023; Ferguson et al., 2019; Fulceri et al., 2016; Kang et al., 2014; Mazefsky et al., 2014; Neuhaus et al., 2018; Prosperi et al., 2017; Restrepo et al., 2020). Other somatic complaints have been associated with GI problems (Ferguson et al., 2019; Fulceri et al., 2016; Mazefsky et al., 2014; Restrepo et al., 2020). Sleep problems were linked to GI problems in most studies (McCue et al., 2017; Neumeyer et al., 2019; Prosperi et al., 2017; Restrepo et al., 2020; Reynolds et al., 2021), though not all (Ferguson et al., 2019). Demographic findings are also inconsistent: GI problems appear unrelated to IQ in autism (Chandler et al., 2013; Jiang et al., 2017; Mazefsky et al., 2014; Mazurek et al., 2013; Prosperi et al., 2017; Restrepo et al., 2020) and are more common in younger children in some studies (Gorrindo et al., 2012; Mazefsky et al., 2014) but not in others (Babinska et al., 2020; Ferguson et al., 2019; Mazurek et al., 2013). GI complaints were more frequent in females in some studies (Babinska et al., 2020; Mazefsky et al., 2014) but not all (Mazurek et al., 2013; Prosperi et al., 2017; Restrepo et al., 2020), and were more common in Caucasian children in one study (Mazurek et al., 2013) but not significantly related to ethnicity in another (Nikolov et al., 2009).

In summary, prior research on demographic, psychological, autistic trait, and somatic correlates of GI problems in autistic youth has produced inconsistent results. Most studies did not control for multiple comparisons, potentially inflating significant findings, and many failed to report effect sizes that might reveal trivial associations. Most relied on univariate analyses (e.g., correlations, *t*-tests), which cannot establish whether a factor is independently related to GI symptoms. The relationship may be due to the variable’s association with another variable that is directly and independently related to GI symptoms. We address these limitations by examining a large sample of autistic children and adolescents across broad age and IQ ranges, testing multiple predictors simultaneously using regression analyses. Our study aims to answer the following research question: What variables are the strongest independent predictors of GI problems when limited food preferences, abnormal sensory processing, autism severity, developmental regression, IQ, oppositional behavior, anxiety, depression, ADHD, somatic, sleep, and demographic variables are entered together in regression analysis?

## Methods

### Procedure

All children (*N* = 1,093) were consecutive referrals to a Department of Psychiatry and Behavioral Health Diagnostic Clinic who were diagnosed with autism and were 2–17 years of age. The Institutional Review Board waived informed consent because our study was a retrospective analysis of existing clinical data. All children underwent a comprehensive diagnostic evaluation by a licensed PhD psychologist with extensive training and experience in diagnosing autism. The evaluation included a semi-structured diagnostic interview with the parents using a validated autism diagnostic instrument (Checklist for Autism Spectrum Disorder [CASD]; Mayes, 2012); questionnaires and rating scales completed by parents and teachers or childcare providers (CASD and Pediatric Behavior Scale [PBS]; Lindgren & Koeppl, 1987); administration of IQ, achievement, and other psychological tests; and clinical observations of the child during psychological testing. All children had a score in the autism range on the CASD and a *DSM*-*IV* or *DSM*-5 diagnosis of autism (i.e., autistic disorder, Asperger’s disorder, or autism spectrum disorder) made by the psychologist conducting the evaluation.

### Instruments

The CASD is a 30-item diagnostic measure completed by a clinician based on a semi-structured parent interview, teacher and child care provider report, and clinical observations of the child during the evaluation. In the nationally representative standardization study, the CASD identified children with and without autism with 99.5% accuracy, and studies show the CASD differentiates autistic children from children who have other diagnoses without autism (Mayes et al., 2012, 2017; Tierney et al., 2015). Diagnostic agreement between the CASD and the Autism Diagnostic Interview-R, Childhood Autism Rating Scale, and Gilliam Asperger’s Disorder Scale ranged from 93% to 98% (Mayes et al., 2009; Murray et al., 2011).

Mothers rated PBS items on a 4-point scale from “almost never or not at all” (0) to “very often” a problem (3) during the past 2 months. The PBS corresponds well with established measures of psychopathology and has been used to diagnose and differentiate psychological and somatic problems in multiple studies (e.g., Conrad et al., 2010; Nichols et al., 2000; Waxmonsky et al., 2017; Wolraich et al., 1994).

### Variables

The four dependent variables were mother ratings on four PBS items: “stomachaches,” “constipated,” “diarrhea or loose bowels,” and “has bowel movements outside of the toilet, soils pants.” The 20 independent variables were PBS and CASD scores and demographics. These included: three dichotomized scores on the CASD (picky eating/limited food preferences, sensory aversion/tactile defensiveness, and developmental regression); total autism symptom score on the CASD; PBS total subscale scores for autism, attention deficit, hyperactive, impulsive, oppositional/aggressive behavior, irritability/tantrums, anxiety, depression, sleep problems, somatic symptoms excluding GI problems (headaches, other aches/ pains excluding stomachaches, complains of feeling sick); age; IQ; sex; race; parent occupation; and psychotropic medication use.

### Data Analyses

Pearson correlations were calculated to determine inter-relationships between the four GI symptom scores (stomachaches, constipation, diarrhea, and bowel incontinence). To examine the relationship between the GI symptom scores and the independent variables, correlations and explained variance were calculated (Pearson correlations for continuous variables and point-biserial correlations for dichotomous variables). Chi-square and the effect size statistic phi were used to examine dichotomized variables. Stepwise regression analysis determined variables that were significant independent predictors of stomachaches, constipation, diarrhea, and bowel incontinence, controlling for the other potentially relevant variables. SPSS was used for statistical analyses.

## Results

### Sample

The sample comprised 1,093 autistic children aged 2–17 years (*M* = 6.5 years, *SD* = 3.3). Full-scale IQs ranged from 9 to 146 (*M* = 91.2, *SD* *=* 25.6). In all, 82.0% were male, 89.3% were White, 34.7% had a parent with a professional or managerial occupation, and 31.8% were treated with psychotropic medication.

### Correlations Between GI Symptoms

Inter-correlations between the four GI symptom scores were weak (Table 1). Stomachaches had a small-to-medium positive significant correlation with constipation (.24, explained variance 5.8%) and diarrhea (.22, explained variance 4.8%). All other correlations between the GI symptoms were small (explained variance 0.0%–3.2%).

**Table 1.** Correlations Between the GI Symptoms Scores.

	Stomachaches		Constipation		Diarrhea	
GI symptom	*R*	*p*	*r*	*p*	*r*	*p*
Constipation	.24	<.01				
Diarrhea	.22	<.01	.01	1.0		
Bowel Incontinence	−.06	.16	.14	<.01	.18	<.01

*Note. p*-values have a Bonferroni correction for the four GI comparisons.

### Correlations Between GI Symptoms and Independent Variables

#### Autism Symptoms and Severity

As shown in Table 2, autism severity and specific autism symptoms potentially related to GI problems (picky eating, sensory hypersensitivity, and developmental regression) were all small and weakly associated with constipation, stomachaches, diarrhea, and bowel incontinence (explained variance 0.0%–2.6%).

**Table 2.** Correlations Between Gastrointestinal Symptoms and Psychological, Somatic, and Demographic Variables in Autistic Youth (*N* = 1,093).

CASD,/PBS scores and demographics	Stomachaches		Constipation		Diarrhea bowel		Incontinence	
	*r*	*p*	*r*	*p*	*r*	*p*	*r*	*p*
CASD scores								
Picky eating	.03	1.0	.11	<.01	.04	.74	.04	.66
Sensory aversion	.12	<.01	.07	.10	.05	.31	.02	1.0
Developmental regression	−.09	.01	−.03	1.0	.02	1.0	.13	<.01
CASD autism score	.07	.07	.06	.22	.12	<.01	.14	<.01
PBS scores								
Autism	.16	<.01	.11	<.01	.11	<.01	.10	<.01
Attention deficit	.15	<.01	.09	.02	.04	.68	.08	.04
Hyperactivity	.07	.11	−.00	1.0	.07	.09	.10	<.01
Impulsivity	.14	< .01	.05	.52	.09	.02	.13	<.01
Oppositional/aggressive	.14	<.01	.06	.12	.06	.15	.09	.01
Irritability/tantrums	.18	< .01	.09	.01	.07	.08	.05	.28
Anxiety	.22	< .01	.11	<.01	.12	< .01	−.02	1.0
Sadness	.35	<.01	.14	< .01	.07	.06	−.06	.26
Sleep problems	.17	< .01	.11	< .01	.10	.01	.09	.02
Somatic symptoms^a^	.64	< .01	.16	< .01	.15	< .01	−.09	.01
Demographics								
Age	.27	< .01	.07	.10	−.04	.77	−.25	< .01
IQ	.12	< .01	.00	1.0	−.04	.87	−.22	< .01
Male	−.05	.30	−.01	1.0	.08	.03	−.00	1.0
White	.06	.21	−.02	1.0	.09	.02	.00	1.0
Professional parent	−.01	1.0	−.06	.18	.02	1.0	−.08	.05
Psychotropic medication use	.18	< .01	.03	1.0	.02	1.0	−.08	.02

*Note. p*-values have a Bonferroni correction for the four GI group comparisons for each independent variable.

a Excluding GI problems.

#### Externalizing Symptoms

The correlations between externalizing symptoms and the four GI symptoms (Table 2) were small (explained variance 0.0%–3.2%). Of the 430 children rated by their mothers as often or very often oppositional/aggressive, 45.5% had bowel incontinence often or very often and 39.0% did not. The difference was not significant (χ^2^ = 0.5, phi = 0.0, *p* = .47).

#### Internalizing Symptoms

The only correlations between the four GI symptoms and the 12 CASD autism and PBS psychopathology scores (Table 2) that were not small were between stomachaches and feeling sad (*r* = .35, explained variance 12.2%) and between stomachaches and feeling anxious (*r* = .22, explained variance 4.8%). All other correlations between anxiety and sadness and the remaining GI symptoms were small (explained variance 0.0%–2.0%). Of the 447 children (40.9%) rated by their mothers as often or very often anxious, 74.3% had stomachaches often or very often and 37.3% did not. Of the 132 children (12.1%) rated as feeling sad often or very often, 37.1% had stomachaches often or very often and 9.4% did not.

#### Somatic and Sleep Problems

The correlation between stomachaches and somatic symptoms excluding GI problems was large (explained variance 41.0%). All other correlations between GI symptoms and sleep problems and somatic problems (excluding GI symptoms) were small (explained variance 0.8%–2.9%).

#### Demographics and Medication Use

Age had a small to medium positive correlation with stomachaches (explained variance 7.3%) and a small to medium negative correlation with bowel incontinence (explained variance 6.2%). Bowel incontinence also had a small to medium negative correlation with IQ (explained variance 4.8%). All other correlations between GI symptoms and demographics (age, IQ, sex, race, and parent occupation) were small (explained variance 0.0%–1.4%). Correlations between psychotropic medication use and the four GI symptoms were also small or small-medium (explained variance 0.0%–3.2%).

### Regression Analysis

The score on somatic symptoms that exclude GI problems was the strongest concurrent predictor of stomachaches (explained variance 41.6%), constipation (explained variance 2.5%), and diarrhea (explained variance 2.4%) in stepwise regression analyses (Table 3). Adding the next strongest predictor from the psychological, demographic, and psychotropic medication use variables increased explained variance by less than 2%. Findings for bowel incontinence were different. The somatic symptom score excluding GI problems was not a significant predictor, whereas younger age followed by lower IQ were significant, explaining 10.6% of the variance. Because of the significance of age and IQ, analyses were repeated with the subsample (*n* = 430) of children ages 6 or older and with an IQ of 80 or higher. The somatic symptom score excluding GI problems was again not a predictor, nor were age and IQ. Oppositional/aggressive behavior was the strongest predictor, but explained only 1.9% of the variance (*F* = 8.4, *p* = .004, *β* = 0.14).

**Table 3.** Significant Concurrent Predictors of GI Symptom Scores Using PBS Psychopathology and Somatic Symptom Scores, Demographics, and Psychotropic Medication Use as Predictor Variables in Regression Analysis.

GI symptom	Significant predictors^a^	*R*	Explained variance^b^	Standardized *β*	*t*
Stomachaches	Somatic symptoms^c^	.64	41.6%	0.64	27.9
Constipation	Somatic symptoms^c^	.16	2.5%	0.16	5.2
Diarrhea	Somatic symptoms^c^	.15	2.4%	0.15	5.1
Bowel incontinence	Age	.25	6.3%	−0.24	8.2
	IQ	.32	10.6%	−0.21	7.2

*Note.* All *t*-values *p* < 0.001. Model summary stomachaches *F* = 776.1, *p* < 0.001; constipation *F* = 27.5, *p* < 0.001, diarrhea *F* = 26.4, *p* < 0.001, bowel incontinence *F* = 64.4, *p* < 0.001.

a *p* < 0.001 and increase in explained variance >2%. ^b^ Cumulative *R* and explained variance including each previous significant predictor. ^c^ Somatic symptom score that excludes GI problems (headaches, other aches/ pains excluding stomachaches, complains of feeling sick).

## Discussion

Inter-correlations between the four GI symptoms (stomachaches, constipation, diarrhea, and bowel incontinence) were less than medium (explained variance 0%–6%), suggesting they are not intrinsically connected to any large degree in autistic children. This finding highlights the necessity of investigating GI problems separately and not only analyzing a combined or composite score.

The strongest concurrent predictor of stomachaches in regression analysis was the somatic symptom score that excluded GI problems. It was also the strongest predictor of constipation and diarrhea, but in both cases, it explained only 2% of the variance. None of the remaining psychological, demographic, and psychotropic medication use variables increased explained variance by 2% or more. These results show that stomachaches are clearly linked with other somatic symptoms, most likely because of their mutual association with physical pain and discomfort. Results suggest that, overall, for autistic children, stomachaches, constipation, and diarrhea may manifest primarily as medical or physiological problems not significantly correlated with psychological variables (except for the significant relationship between stomachaches and sadness and anxiety), although biopsychological interactions may be a factor.

Unlike other GI problems, the somatic symptom score in our study was not a significant predictor of bowel incontinence, whereas younger age, followed by lower IQ, were. However, in the subsample of children > 6 years with an IQ > 80, age and IQ were not significant. Bowel incontinence is often thought to be a behavior problem in autistic children. This may be accurate for a small percentage of children, but not for most, according to our results. Interestingly, information provided by parents of children in our study during the clinical diagnostic interview indicated that many of the children with bowel incontinence held their feces (e.g., delaying or having a bowel movement at home but not at school), resulting in soiling. Others willingly had bowel movements in a pull-up but not in the toilet, and many of these children indicated they were afraid to have a bowel movement in the toilet. Bowel incontinence may be related to sensory aversion and fear and may represent one of the idiosyncrasies of autism that is difficult to explain other than its association with autism. Recommendations for reducing bowel incontinence include a combination of medication, behavior, and dietary management (Wassom & Christophersen, 2014). A small pilot intervention that included suppositories, behavior management focused on rewards, and scheduled toilet sessions in autistic children ages 5–16 showed that 6 of the 10 participants achieved bowel continence by the end of the intervention, compared to none of the 10 in the wait-list control group (Mevers et al., 2020). Future research investigating these multi-component interventions will help to better understand the relationship between autism and bowel incontinence.

The independent variables analyzed in our study yielded weak correlations with GI problems (although often significant, arguably because of the large sample size), and almost all were nonsignificant predictors in regression analysis when other variables were controlled. These variables included, with a few exceptions, externalizing and internalizing symptoms, sleep problems, picky eating, sensory hypersensitivity, demographics, and psychotropic medication use. Thirty of the 80 univariate correlations between the 4 GI symptoms and 20 psychological, autism symptom, somatic, medication, and demographic variables were statistically significant but only 1 of the 30 significant correlations was large (i.e., correlations between stomachaches and non-GI somatic symptoms), 1 was medium (stomachaches and sadness), and 4 were small to medium (stomachaches and anxiety and increasing age and bowel incontinence and decreasing age and decreasing IQ). All other correlations were small, explaining 3% or less of the variance. Further, we did not find a strong combined or cumulative effect of externalizing and internalizing symptoms, sleep problems, picky eating, sensory hypersensitivity, and demographics on GI problems, as together, these variables contributed little to the prediction of GI problems in regression analysis.

Our findings highlight the importance of considering effect sizes (e.g., explained variance) when interpreting the clinical significance of statistically significant results and the importance of conducting multivariate analyses (in addition to univariate analyses) to determine significant independent predictors of GI problems, controlling for other variables. Previous studies reviewed in our Introduction reported both significant and nonsignificant relationships between GI problems and autism severity, picky eating, sensory hypersensitivity, developmental regression, attention deficit, oppositional/aggressive behavior, anxiety/depression, somatic complaints, sleep problems, age, sex, and race. The fact that both significant and nonsignificant results are found supports the weak association between GI problems and these variables. Further, many studies reported in our literature review did not adjust *p*-values to control for multiple comparisons. Common source variance (using the same rater and instrument for assessing psychological and GI problems) may have inflated correlations in some studies, including ours. Last, GI problems and psychopathology may be indirectly related to each other because of their shared association with autism and not because of a causal relationship.

Notably, in univariate analyses, the strongest correlations between psychopathology and GI symptom scores were between stomachaches and sadness and feeling anxious. All other correlations between anxiety and sadness and the remaining GI symptoms were small. This supports previous research showing a relationship between internalizing symptoms and stomachaches (Saps et al., 2009). However, sadness and anxiety increased explained variance for stomachaches by <2% beyond what was explained by other somatic symptoms in regression analysis. Given our findings, intervention for stomachaches should continue to be medically based, but addressing anxiety and depression using evidence-based therapy and psychotropic medication may help reduce stomachaches for those children who are anxious or sad.

Food selectivity is common in autism, and grain products (e.g., pasta, pizza, cereal) and chicken nuggets were the preferred foods for 92% of autistic children who had limited food preferences (Mayes & Zickgraf, 2019). Given autistic children’s preference for grain products, it is unlikely that food selectivity is a major cause of constipation in autism. This is supported by our findings of very small correlations between selective eating and constipation and other GI problems. Interestingly, dietary intervention and food supplements were not found to be effective in decreasing GI problems in 247 autistic children 2–18 years of age (Babinska et al., 2020).

Age had a small to medium positive correlation with stomachaches (.27, explained variance 7%) and a small to medium negative correlation with bowel incontinence (−.25, explained variance 6%). Bowel incontinence also had a small to medium negative correlation with IQ (−.22, explained variance 5%). All other correlations between GI symptoms and demographics (age, IQ, sex, race, and parent occupation) were very small, suggesting that sex, race, and parent occupation are not associated with GI problems in autism.

Although GI symptoms are far more common in autism than in ADHD (Mayes et al., 2021), other developmental disorders (Valicenti-McDermott et al., 2006), and neurotypical children (Mayes et al., 2021; Valicenti-McDermott et al., 2006), autism severity and specific autism symptoms conceivably related to GI problems (picky eating, sensory hypersensitivity, and developmental regression) were only weakly associated with constipation, stomachaches, diarrhea, and bowel incontinence. This suggests that factors other than these traits may better explain the association between autism and GI symptoms. The strongest predictor of GI problems in our study was the presence of other somatic complaints, which could reflect a functional or a medical-biological or hybrid explanation.

A review by Wasilewska and Klukowski (2015) concluded that autism and GI symptoms may have shared pathophysiological pathways (including intestinal inflammation, food allergies, gluten-related disorders, and dysregulation of the gut microbiome) and/or pathogenetic factors (including functional mechanisms such as visceral hypersensitivity and dysautonomia). A recent meta-analysis of GI disorders and autism found that autism was significantly associated with increased odds of inflammatory bowel disease, ulcerative colitis, and Crohn’s disease (Kim et al., 2022). Recent reviews have laid the foundation for future research regarding potentially unique microbiota composition in youth autism (Rose et al., 2018; Yu et al., 2024). A recent study found that autistic children had significantly different abnormal esophageal histology compared to children without autism, but notably not significantly different stomach findings (Ballal et al., 2024). A study by Ferguson et al. (2017) suggested that autonomic function and GI problems are intertwined in autistic children, although causality could not be determined. In addition, a review by Hsiao (2014) hypothesized a gut–brain connection in autistic youth. Recent reviews argue for future research examining the central nervous system’s role in GI symptoms for individuals with autism (Hung & Margolis, 2024; Lefter et al., 2019). Research on biological and genetic links between autism and GI problems is scant, provides mixed conclusions, and often involves very small samples.

### Limitations and Future Directions

Our sample is from a single clinical site, so results need to be replicated in other clinical and nonclinical settings with families from more diverse backgrounds and cultures than those in our sample. We investigated four key GI problems in autism, but future research should investigate additional GI symptoms, including reflux, nausea, bloating, and vomiting. Importantly, our study did not have access to data on whether children were treated for their GI problems (e.g., consulting a GI specialist, using a stool softener, or participating in a bowel management program). Our study included a single variable, psychotropic medication use. Future research should conduct a stratified analysis by medication class of specific psychotropic medications and other medications, examining their possible relationship to GI problems and the variables analyzed in our study. This is a complex but important undertaking because autistic children may be prescribed a variety of medications including psychotropics and medications to treat GI problems and other medical conditions, as well as using nonprescription treatments that may positively and negatively affect GI and psychological symptoms. Importantly, potential medical, physiological, genetic, and dietary variables need to be explored to explain the relationship between autism and GI symptoms. Shared method variance (same rater, same instrument) may have artificially inflated the relationship between GI problems and other variables. Therefore, future studies are needed using independent measures of GI symptoms and potential predictor variables.

## Conclusion

Strikingly, our study found 50 nonsignificant correlations between the 4 GI symptoms and 20 comorbid psychopathology, autism symptom, somatic complaint, and demographic variables and 30 statistically significant correlations, only two of which were medium or large. Effect sizes (explained variance) were generally very small. Furthermore, in multivariate analyses, after controlling for all potential predictor variables, only the non-GI somatic symptom score significantly predicted constipation, stomachaches, and diarrhea. Notably, across studies of the association between psychological, autism, somatic complaint, and demographic variables and GI problems reviewed in our Introduction, both significant and nonsignificant relationships for each independent variable were found, indicating an overall weak association, consistent with our findings. Our findings suggest that researchers and clinicians need to search beyond comorbid psychopathology and demographics and consider other functional, physiological, neurobiological, and genetic possibilities for why GI problems are so common in autism relative to children with other neurodevelopmental disorders and neurotypical children. Coury et al. (2012) maintained in their review of GI symptoms in autism that our limited understanding of the pathology underlying GI symptoms in autism undermines the scientific rationale for many current interventions (e.g., nutritional supplements). A multidisciplinary panel of 27 experts reviewed the medical literature with the goal of generating evidence-based recommendations for diagnostic evaluations and management of GI problems in autistic individuals and concluded that evidence-based recommendations are not yet available (Buie et al., 2010).

The findings of this study have important implications for education, family support, and clinical practice by highlighting that while GI symptoms in autistic children may have behavioral correlates, they are more likely influenced by underlying medical and physiological factors. In educational settings, flexible bathroom policies, sensory-friendly toileting environments, and staff training may help reduce anxiety and stigma surrounding GI issues. For families, results emphasize the importance of seeking medical evaluations for GI symptoms in addition to behavioral interventions, which can alleviate parental guilt and improve the parent–child relationship. Clinicians should incorporate routine screening for GI symptoms, provide psychoeducation to families, and adopt a multidisciplinary approach by making referrals to gastroenterologists when appropriate. These practices can prevent mislabeling GI symptoms as purely behavioral problems and promote more effective, supportive interventions for GI problems in autistic children, enhancing their overall health and the quality of life of the children and their parents.

## Data Availability

Data are not available due to patient confidentiality.
